# Rosmarinic Acid and Its Methyl Ester as Antimicrobial Components of the Hydromethanolic Extract of *Hyptis atrorubens* Poit. (Lamiaceae)

**DOI:** 10.1155/2013/604536

**Published:** 2013-11-17

**Authors:** Amin Abedini, Vincent Roumy, Séverine Mahieux, Murielle Biabiany, Annie Standaert-Vitse, Céline Rivière, Sevser Sahpaz, François Bailleul, Christel Neut, Thierry Hennebelle

**Affiliations:** ^1^Univ Lille Nord de France, 59000 Lille, France; ^2^UDSL, Laboratoire de Pharmacognosie, EA 4481 GRIIOT, UFR Pharmacie, 59000 Lille, France; ^3^UDSL, Laboratoire de Bactériologie, INSERM U995, UFR Pharmacie, 59000 Lille, France; ^4^APLAMEDAROM, Association des Plantes Médicinales et Aromatiques de Guadeloupe, Mompierre, 97111 Morne-à-l'eau, Guadeloupe, France; ^5^INSERM U1019, CNRS UMR 8204, UDSL, Pasteur Institute, Center for Infection and Immunity of Lille (CIIL) EA-4547, Biology & Diversity of Emerging Eukaryotic Pathogens (BDEEP), UFR Pharmacie, 59010 Lille, France

## Abstract

Primary biological examination of four extracts of the leaves and stems of *Hyptis atrorubens* Poit. (Lamiaceae), a plant species used as an antimicrobial agent in Guadeloupe, allowed us to select the hydromethanolic extract of the stems for further studies. It was tested against 46 microorganisms *in vitro*. It was active against 29 microorganisms. The best antibacterial activity was found against bacteria, mostly Gram-positive ones. Bioautography enabled the isolation and identification of four antibacterial compounds from this plant: rosmarinic acid, methyl rosmarinate, isoquercetin, and hyperoside. The MIC and MBC values of these compounds and their combinations were determined against eight pathogenic bacteria. The best inhibitory and bactericidal activity was found for methyl rosmarinate (0.3 mg/mL). Nevertheless, the bactericidal power of rosmarinic acid was much faster in the time kill study. Synergistic effects were found when combining the active compounds. Finally, the inhibitory effects of the compounds were evaluated on the bacterial growth phases at two different temperatures. Our study demonstrated for the first time antimicrobial activity of *Hyptis atrorubens* with identification of the active compounds. It supports its traditional use in French West Indies. Although its active compounds need to be further evaluated *in vivo*, this work emphasizes plants as potent sources of new antimicrobial agents when resistance to antibiotics increases dramatically.

## 1. Introduction

Plants have been used for ages to treat human diseases and it has been estimated that 25 to 50% of currently available drugs are derived from plants [1]. One of the families of drugs with the most urgent need for new members is antibiotics. In 2011, the WHO (World Health Organization) asked for increased search for new drugs as antibiotic resistance increases dramatically, but only few new molecules are in development. Plants can be a source of new antimicrobial drugs since they are considered as time-tested and comparatively safe both for human use and the environment [2].

*Hyptis atrorubens *Poit. is a herb of the Lamiaceae family, the members of which are frequently used for antimicrobial purposes [3]. This species is native to tropical America. Its chemical composition has been little studied, except for the volatile components. It has at least two identified essential oil chemotypes: estragole + limonene (chemotype A) or germacrene D (chemotype B) [4]. In the French West Indies, fresh leaves are used topically against dermatitis and athlete's foot [5]. In French Guiana, infusion or decoction of aerial parts is used against sore throat and flu [6]. The leaves of *H. atrorubens* showed no anti-HIV activity but displayed cytotoxic activity (IC_50_: 25 *μ*g/mL) against a culture of human lymphoblastic leukemia cell line [7]. No experimental data about the antimicrobial activity of this plant were encountered.

The aim of this study was to investigate the antimicrobial activity of *H. atrorubens* against 46 microorganisms including bacteria, yeasts, and dermatophytes, to purify and identify active compounds, and to test the most active ones on the growth of selected bacterial strains.

## 2. Materials and Methods

### 2.1. Chemical Analysis

#### 2.1.1. Materials

Optical rotations were measured on a Perkin-Elmer 343 polarimeter. UV spectra were recorded on a Biochrom WPA Lightwave II UV-visible spectrometer. IR spectra were recorded on a Thermo Nicolet Avatar 320 FT-IR spectrometer. NMR spectra (^1^H: 500 MHz, ^13^C: 125 MHz) were recorded on a Bruker Avance 500 spectrometer in the Laboratoire d'Application de RMN (LARMN), Université de Lille 2. Mass spectra (ESI-MS^2^) were performed on a PE Sciex API 3000 triple quadrupole mass spectrometer equipped with an ion spray turbo source (Perkin-Elmer Sciex). Chromatographic separations were carried out by MPLC on a Büchi system composed of a C-605 pump module and a Büchi column (Büchi 460 × 35 mm packed with Merck Lichroprep RP18 25–40 *μ*m, Darmstadt, Germany). The HPLC apparatus comprised a Shimadzu LC-10AS pump and SCL-10A detector with an analytical and a semipreparative RP18 column (Silica Upti-prep Strategy, 100 Å, 5 *μ*m RP, 25 × 4.6 or 10 mm Interchrom, Interchim, Montluçon, France). TLC was performed on silica gel 60 F_254_ plates (Merck, Darmstadt, Germany).

#### 2.1.2. Plant Materials and Extract Preparation

The stems and leaves of *Hyptis atrorubens* were identified and collected in Gros Morne Diomar, Guadeloupe, in June 2011 by M.B. A voucher specimen is conserved in the French Institute of the Agricultural Research Centre (INRA) at Petit-bourg (Guadeloupe) with herbarium number 10 473. After a preliminary antimicrobial screening of various plant parts by bioautography, stems were selected for further studies and purification of pure compounds. Dried and powdered stems (1 100 g) were extracted by maceration using four successive solvents (5 L) of increasing polarity: petroleum ether, dichloromethane, methanol, and water-methanol (1 : 1, v/v). The extraction was repeated thrice (each time 2 hours). The extracts were filtered and the solvents were removed by evaporation under reduced pressure to give the dry residues. The amounts of extracts were 4.5 g (petroleum ether), 6.1 g (dichloromethane), 61.2 g (methanol), and 80.4 g (water-methanol), respectively.

#### 2.1.3. Purification and Identification of the Compounds

The *H. atrorubens* stem hydromethanolic extract (80.4 g) was dissolved in H_2_O and submitted to a H_2_O-CH_3_OH (0 to 100% CH_3_OH) gradient on Sephadex LH-20. There were nine fractions named A to I. Bioautography showed B, C, and E to be the most active fractions. Fraction B (0.8 g) was submitted to a H_2_O-CH_3_OH (0 to 100% CH_3_OH) MPLC gradient and to semipreparative HPLC [H_2_O/CH_3_OH (4 : 1)] to yield the pure compound RA (746 mg). Fraction C (1.0 g) was submitted to a H_2_O-CH_3_OH (0 to 100% CH_3_OH) MPLC gradient, that afforded a crude mixture of compounds MR and IQ, that was submitted to further purification by HPLC using an increasing amount of CH_3_OH in H_2_O, following the sequence (% CH_3_OH): 20% (isocratic, 15 min), 20 to 40% (linear gradient, 5 min); 40% (isocratic, 10 min) and yielded compounds IQ (35 mg) and MR (20 mg). Fraction E (0.8 g) was submitted to a H_2_O-CH_3_OH (0 to 100% CH_3_OH) MPLC gradient and a subsequent semi-preparative HPLC (H_2_O/CH_3_OH (4 : 1)) enabled the purification of compound HS (29 mg).

#### 2.1.4. Data of Isolated Compounds

*Compound RA*. Yellow amorphous powder; C_18_H_16_O_8_; ESI-MS (negative ion) *m/z* 359 [M-H]^−^; UV *λ*_max⁡_ (CH_3_OH): 221, 330 nm; IR (KBr) (cm^−1^) *ν*_max⁡_: 3382, 1697, 1606, 1522; ^1^H-NMR (CD_3_OD): *δ* 7.52 (1H, d, *J* = 15.5 Hz, H-7′), 7.04 (1H, d, *J* = 2.0 Hz, H-2′), 6.91 (1H, dd, *J* = 8.0, 2.0 Hz, H-6′), 6.75 (1H, d, *J* = 8.0 Hz, H-5′), 6.70 (1H, d, *J* = 2.0 Hz, H-2), 6.69 (1H, d, *J* = 8.0 Hz, H-5), 6.57 (1H, dd, *J* = 8.0, 2.0 Hz, H-6), 6.26 (1H, d, *J* = 15.5 Hz, H-8′), 5.19 (1H, dd, *J* = 10.0, 3.5 Hz, H-8), 3.06 (1H, dd, *J* = 14.5, 5.5 Hz, H-7a), 3.00 (1H, dd, *J* = 14.5, 5.5 Hz, H-7b); ^13^C-NMR (CD_3_OD): *δ* 174.3 (C-9), 169.4 (C-9′), 149.5 (C-4′), 145.7 (C-7′), 145.0 (C-3), 144.9 (C-4), 143.2 (C- 3′), 130.4 (C-1), 128.2 (C-1′), 122.2 (C-6), 121.4 (C-6′), 117.6 (C-2), 116.3 (C-5), 116.1 (C-5′), 115.8 (C-8′), 115.3 (C-2′), 77.8 (C-8), 38.9 (C-7).

*Compound MR.* Yellow amorphous powder; C_19_H_18_O_8_; ESI-MS (negative ion): *m/z* 373 [M-H]^−^; UV *λ*_max⁡_ (CH_3_OH): 207, 217, 330 nm; IR (KBr) (cm^−1^) *ν*_max⁡_: 3397, 1727, 1690, 1605, 1521, 1363, 1282, 1162, 1073, 979; ^1^H-NMR (CD_3_OD): *δ* 7.52 (1H, d, *J* = 15.5 Hz, H-7′), 7.04 (1H, d, *J* = 2.0 Hz, H-2′), 6.91 (H, dd, *J* = 8.5, 2.0 Hz, H-6′), 6.75 (1H, d, *J* = 8.5 Hz, H-5′), 6.71 (1H, d, *J* = 2.0 Hz, H-2), 6.64 (1H, d, *J* = 8.0 Hz, H-5), 6.61 (1H, dd, *J* = 8.0, 2.0 Hz, H-6), 6.27 (1H, d, *J* = 15.5 Hz, H-8′), 5.11 (1H, dd, *J* = 7.5, 5.0 Hz, H-8), 3.72 (3H, s, OCH3), 3.06 (1H, dd, *J* = 14.5, 5.5 Hz, H-7a), 3.00 (1H, dd, *J* = 14.5, 5.5 Hz, H-7b); ^13^C-NMR (CD_3_OD): *δ* 172.1 (C-9), 168.2 (C-9′), 150.1 (C-4′), 148.6 (C-7′), 147.3 (C-3′), 146.1 (C-3), 145.7 (C-4), 128.4 (C-1), 127.5 (C-1′), 123.3 (C-6′), 122.8 (C-6), 117.9 (C-2), 116.9 (C-5), 116.2 (C-5′), 115.3 (C-2′), 114.2 (C-8′), 74.9 (C-8), 52.8 (OCH_3_), 38.0 (C-7).

*Compound IQ*. Light-yellow powder; C_21_H_20_O_12_; ESI-MS (negative ion): *m/z* 463 [M-H]^−^; UV *λ*_max⁡_ (CH_3_OH): 227, 252 nm; IR (KBr) (cm^−1^) *ν*_max⁡_: 3310, 1665, 1607, 1507, 1378, 1465; ^1^H-NMR (CD_3_OD): *δ* 7.70 (1H, d, *J* = 2.0 Hz, H-2′), 7.57 (1H, dd, *J* = 7.5, 2.0 Hz, H-6′), 6.85 (1H, d, *J* = 8.0 Hz, H-5′), 6.26 (1H, d, *J* = 2.0 Hz, H-8), 6.10 (1H, d, *J* = 2.0 Hz, H-6), 5.01 (1H, d, *J* = 7.7 Hz, H-1′′), 3.70 (1H, dd, *J* = 12.0, 2.4 Hz, H-6′′a), 3.56 (1H, dd, *J* = 12.0, 5.4 Hz, H-6′′b), 3.47 (1H, dd, *J* = 9.5, 7.7 Hz, H-2′′), 3.42 (1H, t, *J* = 9.0 Hz, H-3′′), 3.34 (1H, t, *J* = 9.0 Hz, H-4′′), 3.21 (1H, dd, *J* = 9.0, 2.4 Hz, H-5′′); ^13^C-NMR (CD_3_OD): *δ* 178.9 (C-4), 167.3 (C-7), 163.2 (C-5), 158.6 (C-9), 158.0 (C-2), 149.9 (C-3′), 149.6 (C-4′), 135.1 (C-3), 123.0 (C-1′), 122.8 (C-6′), 117.4 (C-5′), 116.3 (C-2′), 105.2 (C-10), 101.4 (C-1′′), 99.7 (C-6), 95.41 (C-8), 77.6 (C- 5′′), 76.8 (C-3′′), 74.3 (C-2′′), 70.3 (C-4′′), 61.4 (C-6′′).

*Compound HS*. Yellow needles; C_21_H_20_O_12_; ESI-MS (negative ion): *m/z* 463 [M-H]^−^; UV *λ*_max⁡_ (CH_3_OH): 228, 254 nm; IR (KBr) (cm^−1^) *ν*_max⁡_: 3300, 1660, 1610, 1510, 1376, 1460; ^1^H-NMR (CD_3_OD): *δ* 7.97 (1H, d, *J* = 2.2 Hz, H-2′), 7.54 (1H, dd, *J* = 8.4, 2.2 Hz, H-6′), 6.94 (1H, d, *J* = 8.4 Hz, H-5′), 6.48 (1H, d, *J* = 2.2 Hz, H-8), 6.28 (1H, d, *J* = 2.2 Hz, H-6), 5.01 (1H, d, *J* = 7.7 Hz, H-1′′), 3.77 (1H, dd, *J* = 4.4, 3.7 Hz, H-4′′), 3.64 (1H, dd, *J* = 9.5, 7.7 Hz, H-2′′), 3.64 (1H, d, *J* = 5.5 Hz, H-6′′b), 3.60 (1H, d, *J* = 5.5 Hz, H-6′′a), 3.52 (1H, dd, *J* = 9.5, 4.4 Hz, H-3′′), 3.48 (1H, m, H-5′′); ^13^C-NMR (CD_3_OD): *δ* 177.6 (C-4), 164.1 (C-7), 161.2 (C-5), 159.2 (C-2), 156.3 (C-9), 148.3 (C-4′), 144.7 (C-3′), 133.7 (C-3), 121.8 (C-6′), 121.3 (C-1′), 116.2 (C-5′), 115.3 (C-2′), 103.9 (C-10), 102.3 (C-1′′), 98.6 (C-6), 93.5 (C-8), 75.7 (C- 5′′), 73.4 (C-3′′), 71.3 (C-2′′), 68.1 (C-4′′), 60.6 (C-6′′).

#### 2.1.5. Quantitative Analysis of the Compounds by HPLC

This method was used to measure the amount of each compound in the hydromethanolic extract. 20 *μ*L of five concentrations of each isolated compound and the total extract (5 mg/mL) were injected successively. The mobile phase consisted of two components: a 0.05% solution of acetic acid (solvent A) and acetonitrile (solvent B). The elution gradient used was 17%, 25%, and 50% solvent B at times zero, 30, and 40 minutes, respectively. The flow rate was 1 mL/min. Linearity was verified by obtaining five point calibration curves over the concentration range of 0.006 to 1 mg/mL.

### 2.2. Microbiological Analysis

#### 2.2.1. Culture and Preparation of Microorganisms

The 36 bacteria used in this study (see Table 1) were incubated overnight at 37°C in tubes containing sloping Mueller-Hinton (MH) agar medium. Ten yeasts and dermatophyte clinical isolates were obtained from the collection of the BDEEP Laboratory. They were incubated on Sabouraud agar medium at 37°C for 48 hours or 30°C for 2 weeks, respectively. Then, the bacteria were diluted with Ringer's Cysteine solution (RC) to 10^6^ bacteria/mL by means of serial dilution just before the antimicrobial assays. Final concentration of each bacterial suspension for MIC/MBC determination and bioautography was 10^4^ bacteria/mL. The same process was performed for the fungi.

#### 2.2.2. MIC Determination (Solid Media)

**Figure 1:**
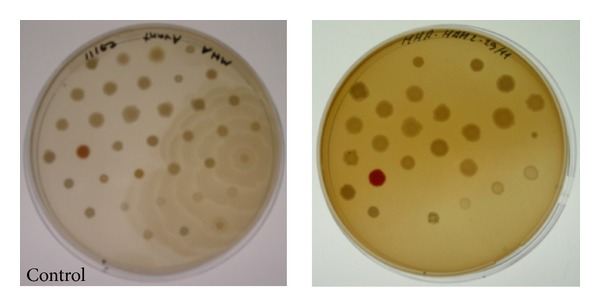
Comparison between the positive control and the hydromethanolic extract of stems at 1.2 mg/mL and 36 bacteria after 24 hours of incubation.

The minimum inhibitory concentration (MIC) was studied using MH agar in Petri dishes seeded by a multiple inoculator [8]. The hydromethanolic extract was tested at six final concentrations (10, 5, 2.5, 1.2, 0.6, and 0.3 mg/mL) against 46 microorganisms. The agar plates were incubated for 24 hours at 37°C. The activity was then estimated visually by the presence or absence of colonies (Figure 1). MIC values were recorded as the lowest concentrations of compounds showing no growth. Solvents used were checked for absence of antibacterial activity. Positive controls were used for bacteria (see Table 1).

#### 2.2.3. Bioautography

To identify the compounds responsible for the antibacterial activity, we adopted an immersion bioautography method [9]. After thin layer chromatography (TLC) (silica gel 60 F_254_, Merck), the plates were covered by MH agar containing a *Staphylococcus epidermidis* 5001 suspension in square Petri dishes. After incubation (24 h at 37°C), growth was revealed by iodonitrotetrazolium chloride (INT) (2 mg/mL) and growth inhibition zones were measured.

#### 2.2.4. MIC/MBC Determinations by Broth Microdilution Method

A serial dilution technique using 96-well microtiter plates was used to determine the MIC of the pure compounds against sensitive bacteria selected by low MIC values [10]. Nine concentrations of each compound, from 2.5 mg/mL to 0.93 × 10^−2^ mg/mL, were used. They were serially twofold diluted with RC in nine wells. Two wells were represented as bacteria culture control (positive control) and medium sterility control (negative control). Then the wells were loaded with MH liquid medium and bacterial suspension (10^4^ bacteria/mL) giving a final volume of 200 *μ*L. The plates were incubated overnight at 37°C. Bacterial growth was indicated visually and then by direct spray of 0.2 mg/mL INT to each well and the plates were incubated at 37°C for at least 30 min. Bacterial growth in the wells was indicated by a reddish-pink color. MIC values were determined as the lowest concentrations of compounds showing clear wells.

The minimal bactericidal concentration (MBC) was determined by the subculture of 100 *μ*L from samples with no visible bacterial growth before INT spray. After 24 hours of incubation, the lowest concentration subcultured showing no growth was considered as the MBC. The isolated substances were also tested together (at equal concentrations) to search for synergy which was evaluated by the FIC (fractional inhibitory concentration) index. The combined effect was calculated by the following formula and results were interpreted as synergy (S, FIC ≤ 0.5), addition (A, 0.5 < FIC < 1), indifference (I, 1 < FIC < 2) and antagonism (AN, FIC ≥ 2) [11].

FIC = (MIC of A in combination/MIC of A alone) + (MIC of B in combination/MIC of B alone).

#### 2.2.5. Time Kill Study

To determine the time-dependent rate of reduction of the bacterial population by active compounds at room temperature, 24-well microtiter plates were used [12]. The surviving population was counted after exposure to two concentrations of pure compound (MIC and 4 × MIC) after five different delays (0, 15 and 60 minutes, and 4 and 24 hours) and compared to controls. Three tubes were prepared (control, MIC, and 4 × MIC) each containing 8 mL of RC. For *T*_0_, 1 mL of bacterial suspension (10^6^ bacteria/mL) was added to every tube. Then 1 mL of RC was added in the control tube, whereas 1 mL of the corresponding concentration of compound was used for the other tubes. 100 *μ*L of tenfold dilutions in RC diluent were plated on MH agar after the different delays indicated above. Colonies were counted after 24 hours of incubation at 37°C. Results are expressed as log CFU/mL.

#### 2.2.6. Growth Curves

In order to follow the growth of bacteria at subinhibitory concentrations of the active compounds at 37°C [13], 4 tubes (control, MIC, MIC/2, and MIC/4) were prepared, each containing 8 mL of Brain Heart liquid medium (BH), 1 mL of bacterial suspension, and 1 mL of the corresponding compound. Tubes were incubated at 37°C. 100 *μ*L of each tube were sampled after 2, 4, 5, 6, 7, 8, and 24 hours, 100 *μ*L of tenfold dilutions were plated on MH agar. Colonies are counted after incubation at 37°C for 24 hours and counts are expressed as log CFU/mL.

#### 2.2.7. Comparison of Growth Curves at 4°C and 37°C

Growth curves were also performed at 4°C, using the same method as described above, to compare the action of the active products on bacteria in growth or stationary phase. 

## 3. Results

### 3.1. MIC Determination of Crude Extracts

Antimicrobial assays were performed on the petroleum ether, methylene chloride, methanolic, and hydromethanolic extracts from the stems or leaves of *H. atrorubens*. Thus, eight extracts were screened in order to select an extract with both good activity and a reasonable probability of recovering sufficient amounts of active compounds for further assays. Results obtained with the stem hydromethanolic extract, which was the most active on a broad spectrum of bacteria, are presented in Table 1. It was active at different degrees against 29 out of 46 microbial strains. *Staphylococcus epidermidis* 10282, *Staphylococcus epidermidis* 5001, *Enterococcus faecalis* C159-6, and *Stenotrophomonas maltophilia* were the most sensitive strains. The MIC of this extract against these four strains was at least 0.3 mg/mL.

The stem hydromethanolic extract was mainly active against Gram-positive bacteria. No antimicrobial activity was found against the following 17 strains: *Citrobacter freundii* 11041; 11042; 11043; *Enterobacter aerogenes* 9004; *Enterobacter cloacae* 11050; 11051; 11053; *Escherichia coli* 8137; 8138; 8157; *Escherichia coli *ATCC 25922; *Klebsiella pneumoniae* 11016; 11017; *Salmonella *sp. 11033; 11037; *Serratia marcescens *11056; 11057; which are all Gram-negative bacteria belonging to the Enterobacteriaceae family. The *in vitro* antifungal activity was also assessed on 5 yeast strains and 5 dermatophytes. The best results were obtained for three dermatophytes (*Trichophyton rubrum, Trichophyton mentagrophytes, *and *Trichophyton tonsurans*). Yeasts were less sensitive than dermatophytes. Given the relatively weak antifungal activity and time-consuming experiments required for dermatophytes, we focused our study only on antibacterial activity for the following steps of the work. 

### 3.2. Purification, Isolation, and Identification

According to these preliminary results (Table 1) and the high extraction efficiency, the hydromethanolic extract of stems was selected and used for further phytochemical studies. The bioautography analysis was performed using *Staphylococcus epidermidis* 5001, one of the most sensitive microorganisms. The results showed optimal activity for fractions B and C and a milder activity for fraction E (Table 2).

**Figure 2:**
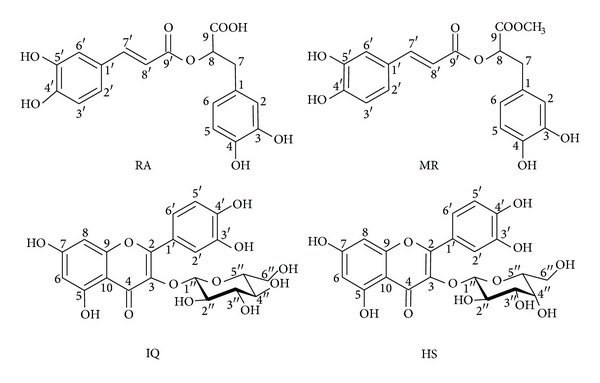
Chemical structure of four active compounds: rosmarinic acid (RA), methyl rosmarinate (MR), isoquercetin (IQ), and hyperoside (HS).

These fractions led to pure compounds *via* bioguided fractionations (see Section 2). Four compounds were detected as responsible for the major part of the antimicrobial activity of *H. atrorubens*. Their structures were established by spectroscopic analysis (mono- and bidimensional NMR, MS) and direct comparison with published data [14, 15] as rosmarinic acid (RA, 0.93% yield), methyl rosmarinate (MR, 0.02%), quercetin-3-O-glucoside (isoquercetin, IQ, 0.04%), and quercetin-3-O-galactoside (hyperoside, HS, 0.03%) (Figure 2). These compounds are known but to the best of our knowledge this is the first report of their presence in the species *Hyptis atrorubens*.

### 3.3. Quantitative Analysis

In order to enable a correct characterization of the assayed extract, all four major active compounds were quantified in *H. atrorubens* stem hydromethanolic extract. The results of quantitative analysis are shown in Table 3. The compound RA (rosmarinic acid) was found in a far higher amount (5.60%) than the three others, which makes *Hyptis atrorubens* a good source of RA.

### 3.4. Antibacterial Activity of the Compounds

The antibacterial compounds were evaluated using microdilution assays against eight bacterial strains to measure the MIC and MBC (Table 4). The MICs ranged from 0.3 mg/mL to the limit concentration of 2.5 mg/mL. At a glance, phenylpropanoids RA and MR clearly display a stronger activity than flavonoids IQ and HS: MIC values of both later compounds were never <1.2 mg/mL, and they were often at the highest assayed concentration of 2.5 mg/mL or above. MR was considered to be the most active compound because it displayed significantly lower MIC and MBC values in most cases, but RA was more bactericidal in 2 cases: *Staphylococcus epidermidis* 5001 and *Stenotrophomonas maltophilia.* The three strains *Staphylococcus epidermidis* 5001, *Stenotrophomonas maltophilia,* and *Enterococcus faecalis* C159-6 displayed the highest sensitivity in this test.

### 3.5. Synergistic Effects between the Compounds

As MIC values of isolated compounds were not so different from those of the crude extract, MICs were also established for mixtures of two or more of these compounds, using the determination of the FIC index (Table 5) to reveal possible synergistic mixtures. Such synergism, although rare, was encountered in a few cases. 

Association of both flavonoids proved, at best, indifferent. A mixture of both phenylpropanoids could result in indifferent or additive effects in most cases. Synergistic actions were mainly observed for the association of at least one phenylpropanoid with at least one flavonoid. The lowest MIC (0.07 mg/mL) was reached with an association of all four compounds, on *Enterococcus faecalis* C159-6.

### 3.6. Bactericidal Curves (Time Kill Study)

**Figure 3:**
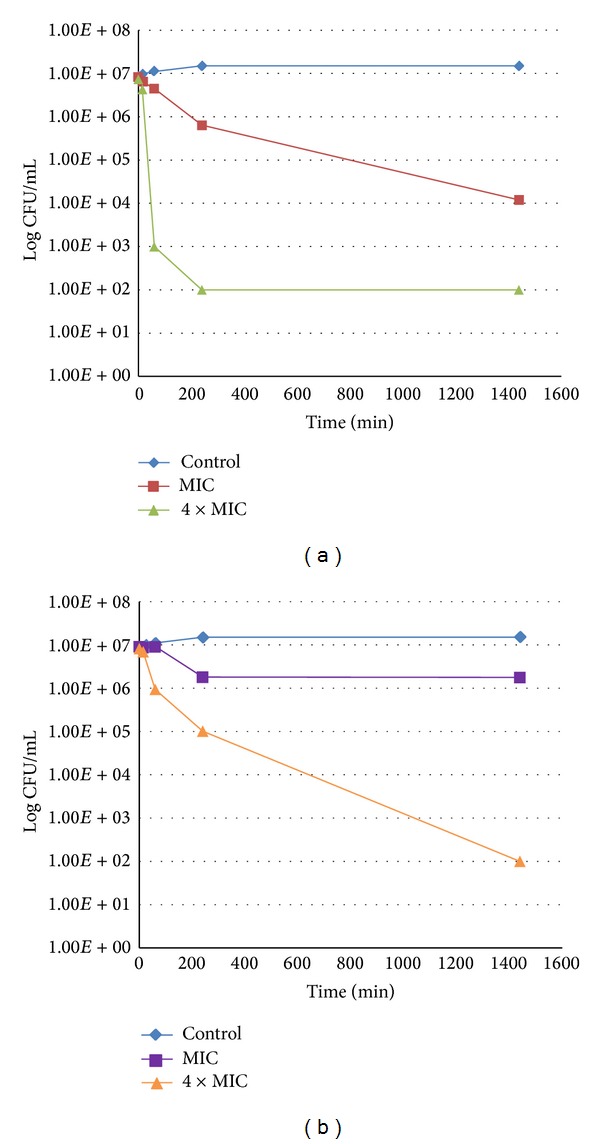
Killing curve of *Staphylococcus epidermidis* 5001 for RA (a) and MR (b).

**Figure 4:**
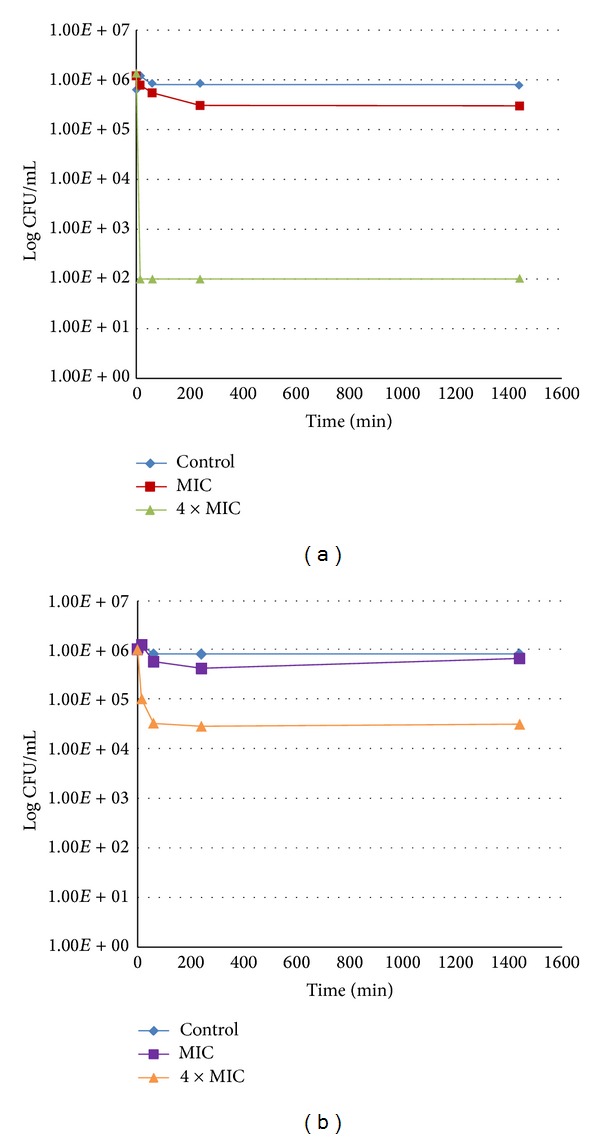
Killing curve of *Stenotrophomonas maltophilia* for RA (a) and MR (b).

In order to establish the time-dependency of the bactericidal action, we measured the rate of reduction of the bacterial population by the two most active compounds (RA and MR) against two bacterial strains: *Staphylococcus epidermidis* 5001 (Figure 3) and *Stenotrophomonas maltophilia* (Figure 4). 

#### 3.6.1. *Staphylococcus epidermidis 5001*

*Staphylococcus epidermidis* 5001 was selected as a model bacterium for Gram-positives. Although *Enterococcus faecalis* C159-6 could be considered as more representative (because it was more sensitive to MR, as was the general case) and more sensitive, we preferred to use *Staphylococcus epidermidis* 5001, which provokes skin infections that are more in accordance with the traditional use of the plant and which is particularly sensitive to RA (the major component of the assayed extract).

A concentration of 1.25 mg/mL (4 × MIC) of RA reduced the bacterial population by 5 log in 4 hours, reaching the detection threshold (<100 bacteria/mL) quickly, whereas MR only reached this threshold after 24 hours. At 0.3 mg/mL (MIC) a similar reduction was observed for both phenylpropanoids after 4 hours, but the decrease in the bacterial population continued only with RA to reach a 3 log reduction after 24 hours.

#### 3.6.2. *Stenotrophomonas maltophilia*

In the case of *Stenotrophomonas maltophilia*, a Gram-negative bacterium, in spite of similar MIC and MBC values of RA and MR in comparison with *Staphylococcus epidermidis* 5001, the difference between the two phenylpropanoids was greater. MR did not reduce the bacterial population by more than 1.5 log at any concentration. In contrast, using RA at 1.25 mg/mL (4 × MIC), bacteria were below the detection threshold (<100 bacteria/mL) after 15 minutes (Figure 4).

### 3.7. Growth Curves

#### 3.7.1. Growth Curves at 37°C

**Figure 5:**
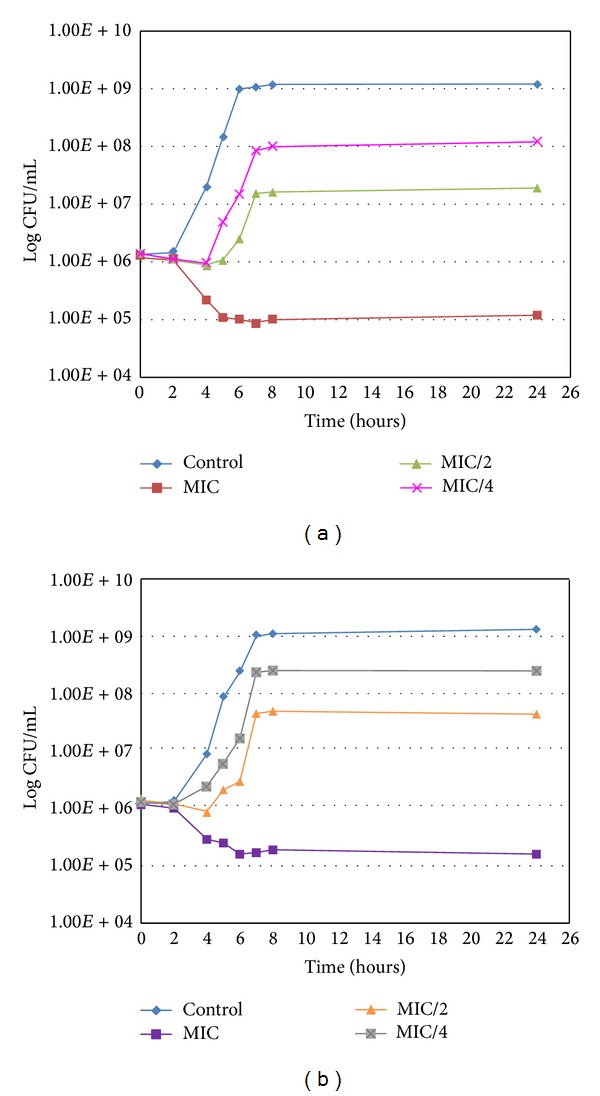
Growth curve of *Staphylococcus epidermidis* 5001 for RA (a) and MR (b) at 37°C.

The three concentrations of RA and MR were tested for their effect on the growth phases of *Staphylococcus epidermidis* 5001 at 37°C. The results are presented in Figure 5.

At the MIC concentration, the two compounds lowered slowly the number of viable bacteria in accordance with the time kill study. At subinhibitory concentrations, the phase of latency was longer and the bacterial level did not reach the control values after 24 hours. Thus, subinhibitory concentrations retained a strong effect on bacterial growth. They delayed the entry of bacteria into the exponential phase: 2 hours at MIC/4 (75 mg/L) and 3 hours at MIC/2 (150 mg/L), for RA. In addition, their stationary phases were one and two log under control, respectively. In Figure 5(b), similar although slightly weaker results are presented for MR.

#### 3.7.2. Growth Curves at 4°C

**Figure 6:**
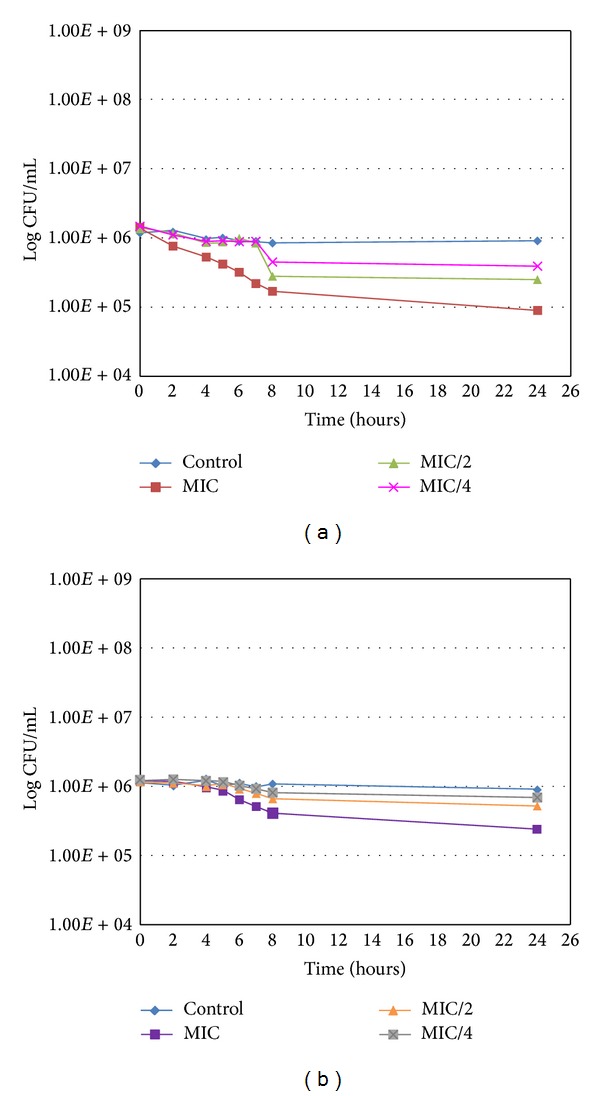
Growth curve of *Staphylococcus epidermidis* 5001 for RA (a) and MR (b) at 4°C.

At this temperature, when the bacteria remained in stationary phase, subinhibitory concentrations had no intense effects on bacterial populations (the maximal reduction observed was by 0.5 log). However, as can be observed in Figure 6(a), the MIC concentration of RA (0.3 mg/mL) reduced the number of bacteria by one log after 24 hours. This result, as can be seen by comparing Figures 5 and 6, is very close to that obtained at 37°C, which means that the antibacterial activity does not need multiplication of the bacteria and is present at the same level in bacteria in stationary phase.

## 4. Discussion

Biological *in vitro* activities of plants should always be submitted to caution. Many studies use very high, nonphysiological concentrations of drugs which will never be achieved in human tissue [16]. The research of antibacterial activity is often only done by diffusion tests which cannot be quantified. Moreover, with that method, different products cannot be compared because the diffusion depends on the molecular size and can be very different from one product to another or be of uncertain significance in crude extracts containing a mixture of different potentially active compounds. 

Antimicrobial studies often concern only a few strains from one or two species. We chose to determine the MIC against a large choice of microorganisms, which enables us to discover extracts with a large-scale activity. Most of the strains have been recently isolated from human infections. For comparison, we also included some reference strains from the American Type Culture Collection (ATCC). These strains, however, have often been isolated years ago and subcultured on laboratory culture medium only and no longer reflect what is encountered today in hospitals. 

 The aim of our study was not only to test crude extracts but also to purify chemically defined compounds. Their biological activities were further characterized. The first approach was done by time kill study which exhibited their time dependent action. Our main compound, RA, decreased the viable count by more than 5 log in a very short time. This action can be compared to that of disinfectants and is much faster than most antibiotics. 

Then growth curves were established, showing that concentrations below the MIC could still slow the growth of the bacterial strains. Even when the concentration was below the MIC level, a biological effect (bacteriostatic effect lasting some hours) can still be present. The growth curves were established by using the time and material consuming method of viable counts. Optical density (OD) is often preferred [17] but many bacterial strains (including *Staphylococcus* and *Pseudomonas *ones) form extracellular compounds increase the OD without changes in viable count. At MIC/2 and MIC/4, our growth curves show both a lag phase before reaching the exponential phase and a lower final concentration. Even after 24 hours, the count remains below the control level (Figure 5). 

The antimicrobial activity of Lamiaceae species is often attributed to volatile components from their essential oil. Although these compounds have been more thoroughly studied, it is often difficult to correlate *in vitro *findings with ethnopharmacological observations as their presence in traditional modes of preparation are often uncertain due to high variability and inadequate extraction protocols. The case of thyme (*Thymus vulgaris* L.) is well known, with at least 7 chemotypes, among which only one has thymol as the major compound [18]. Similarly, the genus *Hyptis* comprises several species that have at least two chemotypes [4]. Consequently, it seemed interesting to test nonvolatile fractions, the composition of which is often less variable, leading to more trustful results. RA is a therapeutic and cosmetic secondary metabolite with very low toxicity. It has already been characterized for many biological activities [19], but its antimicrobial activity has been neither systematically addressed nor quantified. Its mechanism of action is not clearly known in spite of scanning electron micrographs that show a damaged cell surface under RA treatment [20]. The results of our study confirmed and measured the rate of inhibitory and bactericidal activities of this compound against eight pathogenic bacteria, three of which are often concerned by resistance to available antibiotics (*Staphylococcus epidermidis* 5001, *Stenotrophomonas maltophilia,* and *Enterococcus faecalis* C159-6). 

The methyl ester of RA (methyl rosmarinate) displays similar biological activities as the free acid [21]. A recent paper afforded preliminary (MIC on five species) data about its antimicrobial activity [22]. Our study demonstrates that this compound is more inhibitory and bactericidal in comparison with RA. 

Two other compounds, IQ and HS (3-O-glycosides of the flavonol quercetin), are largely present in medicinal plants and food. In spite of strong structural similarity, they differ in their biological activity and bioavailability because they have distinct sugar moieties [23]. These compounds have been studied for numerous activities, among them antiviral [24] and antimicrobial [25, 26].

We tried to measure the antibacterial activity of all four compounds with microbiological indicators (MIC, MBC, and FIC). Although these indicators did not exhibit remarkable activity for either compound (the MIC of RA, for instance, was rather disappointing in comparison with that of the total extract), they did display synergistic effects; stronger inhibitory and bactericidal activity were obtained for mixtures of compounds in six cases and the lowest MIC (70 *μ*g/mL) was achieved for a combination of all four compounds; which is very close to MICs of antibiotics.

 This last observation underlines the importance of the analysis of mixtures of compounds. Synergy studies are seldom done in ethnopharmacology, but seem as a sensible approach, as several different compounds are always found in plants and these associations can contribute to an antimicrobial defense scheme. This needs to be explored [27]. In the crude extract of *H. atrorubens* probably more than the four chemically defined compounds are present. In Table 2, various activities are observed for the fractions E to I which are not further studied here. These fractions will be further analysed to isolate their active compounds. 

Work on the isolated compounds needs to continue in order to reveal their mechanism of action. Electron microscopy can help to visualize cell damage after contact of the bacteria with the identified compounds. Proteomics of the bacterial membrane will reveal modifications after contact. *In vivo* infectious animal models can also be a helpful tool in approaching the practical use of the products. These animal models should focus on infections with frequent implications of Gram-positive microorganisms, as Gram-negatives like enterobacteria are not covered by the identified compounds. Skin infections seem as a convenient target as staphylococci are the most frequently implicated and it is consistent with some traditional uses of the species.

In the case of RA, the presence of a carboxylic acidic group makes it possible that the ionisability of this chemical function and/or the formation of salts with mineral cations is involved, as was observed with antibiotics such as polymyxins [28], bacitracin [29], and daptomycin [30]. This could contribute to explaining discrepancies between the respective activities of this compound and its less acidic methyl ester. 

 In conclusion, our research suggests the presence of up to now underestimated substances with antimicrobial action in natural products. This action is evidenced by different concordant tests like MIC/MBC determination, time kill, growth curves, and synergy tests. Plants have evolved throughout the time in the presence of bacteria and show good resistance against bacterial invasion. They constitute a potential source to strengthen the fight of humans against bacteria that are more and more resistant to antibiotics.

 A part of the results have been published as a poster in the International Congress on Natural Products Research in New York City (June 2012) [31].

## Figures and Tables

**Table 1:** Antimicrobial activity of *Hyptis atrorubens* hydromethanolic extracts (stems and leaves) against 29 microorganisms (*no growth).

	Strains	Stem extract (mg/mL)						Antibiotics		
		10	5	2.5	1.2	0.6	0.3	G	V	A
Gram-negative bacteria	*Acinetobacter baumannii* 9010	∗	∗					S	R	R
	*Acinetobacter baumannii* 9011	∗	∗	∗				R	R	R
	*Proteus mirabilis* 11060	∗	∗	∗				S	R	R
	*Proteus mirabilis* 11061	∗	∗					S	R	R
	*Providencia stuartii* 11038	∗						S	R	S
	***Stenotrophomonas maltophilia***	∗	∗	∗	∗	∗	∗	S	R	R
	*Pseudomonas aeruginosa *8131	∗	∗	∗				S	R	R
	*Pseudomonas aeruginosa ATCC *27583	∗	∗	∗	∗			R	R	R

Gram-positive bacteria	*Mycobacterium smegmatis* 5003	∗	∗	∗	∗	∗		S	S	S
	*Staphylococcus aureus* 8146	∗	∗	∗	∗			S	S	S
	*Staphylococcus aureus* 8147	∗	∗	∗	∗			S	S	S
	***Staphylococcus epidermidis* 10282**	∗	∗	∗	∗	∗	∗	S	S	S
	***Staphylococcus epidermidis* 5001**	∗	∗	∗	∗	∗	∗	S	S	S
	*Corynebacterium *T25-17	∗	∗	∗	∗	∗		S	S	S
	*Enterococcus *sp. 8152	∗	∗	∗	∗			I	S	S
	*Enterococcus *sp. 8153	∗	∗	∗	∗			R	S	S
	***Enterococcus faecalis *C159-6**	∗	∗	∗	∗	∗	∗	R	R	R
	*Staphylococcus lugdunensis *T26A3	∗	∗	∗	∗	∗		S	S	S
	*Staphylococcus warneri *T12A12	∗	∗	∗	∗	∗		S	R	S

Yeast	*Candida krusei *	∗	∗	∗	∗	∗		—	—	—
	*Candida glabrata *	∗						—	—	—
	*Candida kefyr *	∗	∗					—	—	—
	*Candida albicans *	∗						—	—	—
	*Candida parapsilosis *	∗	∗	∗				—	—	—

Dermatophyte	*Microsporum canis *	∗	∗	∗	∗			—	—	—
	*Trichophyton rubrum *	∗	∗	∗	∗	∗		—	—	—
	*Trichophyton mentagrophytes *	∗	∗	∗	∗	∗		—	—	—
	*Trichophyton soudanense *	∗	∗	∗				—	—	—
	*Trichophyton tonsurans *	∗	∗	∗	∗	∗		—	—	—

Microorganisms that were resistant to all extracts are not shown. Positive controls: MIC (*μ*g/mL) gentamicin (G) S: ≤4, R: >8; vancomycin (V) S: ≤4, R: >16; amoxicillin (A) S: ≤4, R: >16.

**Table 2:** Results of bioautography test of fractions “A” to “I”.

Fractions	*S. epidermidis* 10282	*S. epidermidis* 5001	*E. faecalis* C159-6	*S. maltophilia *
A	++	+	+	++
B	+++	+++	++	+++
C	+++	+++	++	+++
D	−	−	−	−
E	+	+	+	−
F	+	+	++	+
G	+	+	+	−
H	+	++	+	−
I	+	++	+	++

(−): no effect, (+): significant effect, rated from + (1 cm inhibition zone) to +++ (>3 cm inhibition zone).

**Table 3:** Results of quantitative analysis of the four compounds by HPLC and linearity of calibration.

Compounds	Retention time (min)	Linear range (mg/mL)	*y* = *ax* + *b* (linear model)	Correlation coefficient (*R* ^2^)	Percent in total extract (%)
RA	20.67 ± 0.45	0.125–1.000	5898776 = (2 × 10^−7^)*x* + 243823	0.9996	5.60
MR	38.77 ± 0.52	0.030–1.000	705509 = (2 × 10^−7^)*x* + 38893	0.9995	0.03
IQ	15.38 ± 0.96	0.006–0.500	103118 = (2 × 10^−7^)*x* − 31067	0.9999	0.08
HS	17.88 ± 0.85	0.006–0.500	218757 = (2 × 10^−7^)*x* − 57439	0.9995	0.06

*y*: peak area of compound in total extract, *x*: concentration of compound in total extract.

**Table 4:** MIC and MBC values in the microdilution assay for the active compounds expressed in mg/mL.

Bacteria	RA		MR		IQ		HS	
	MIC	MBC	MIC	MBC	MIC	MBC	MIC	MBC
*Staphylococcus epidermidis* 5001	0.3	0.3	0.3	0.6	1.2	1.2	2.5	>2.5
*Stenotrophomonas maltophilia *	0.3	0.3	0.3	0.6	1.2	2.5	1.2	1.2
*Enterococcus faecalis C159-6 *	0.3	0.6	0.3	0.3	1.2	2.5	2.5	>2.5
*Staphylococcus lugdunensis T26A3 *	0.6	1.2	0.6	0.6	2.5	2.5	2.5	>2.5
*Pseudomonas aeruginosa ATCC 27583 *	2.5	>2.5	1.2	2.5	1.2	>2.5	2.5	>2.5
*Corynebacterium *T25-17	2.5	>2.5	1.2	1.2	1.2	>2.5	2.5	>2.5
*Mycobacterium smegmatis* 5003	1.2	2.5	0.6	0.6	2.5	>2.5	2.5	>2.5
*Staphylococcus warneri *T12A12	1.2	2.5	0.3	0.3	1.2	>2.5	2.5	>2.5

**Table 5:** Antibacterial activities, indicated by Fractional Inhibitory Concentrations of combined compounds against selected microorganisms.

Bacteria	MIC (mg/mL)-FIC						
	RA + MR	RA + IQ	RA + HS	MR + IQ	MR + HS	IQ + HS	RA + MR + IQ + HS
*Staphylococcus epidermidis* 5001	0.3-(I)	1.2-(AN)	0.3-(I)	0.3-(I)	0.3-(I)	2.50-(I)	0.15-(I)
*Stenotrophomonas maltophilia *	0.3-(I)	0.6-(I)	0.6-(I)	0.3-(I)	0.6-(I)	2.50-(A)	0.15-(I)
*Enterococcus faecalis C159-6 *	0.15-(A)	0.6-(I)	0.3-(I)	0.15-(A)	0.3-(I)	1.25-(I)	0.07-(S)
*Staphylococcus lugdunensis T26A3 *	0.6-(I)	0.3-(A)	0.6-(I)	0.15-(S)	0.6-(I)	2.50-(I)	0.3-(I)
*Pseudomonas aeruginosa ATCC 27583 *	1.2-(I)	1.2-(I)	1.2-(A)	1.2-(I)	1.2-(I)	2.50-(I)	0.6-(I)
*Corynebacterium *T25-17	0.3-(S)	0.6-(A)	0.6-(A)	0.15-(S)	0.6-(A)	1.25-(I)	0.3-(A)
*Mycobacterium smegmatis* 5003	0.3-(A)	0.6-(A)	0.3-(S)	0.3-(A)	0.3-(A)	2.50-(I)	0.3-(A)
*Staphylococcus warneri *T12A12	0.15-(A)	0.6-(A)	0.3-(S)	0.3-(I)	0.3-(I)	2.50-(I)	0.3-(I)

Synergy (S, FIC ≤ 0.5), addition (A, 0.5 < FIC < 1), indifference (I, 1 < FIC < 4), and antagonism (AN, FIC ≥ 4) (ratio 1 : 1).
